# Bacterial Microleakage at the Implant-Abutment Interface: An In Vitro Study

**DOI:** 10.3390/bioengineering9070277

**Published:** 2022-06-26

**Authors:** Simonetta D’Ercole, Tatiane Cristina Dotta, Marzieh Ramezani Farani, Niloofar Etemadi, Giovanna Iezzi, Luca Comuzzi, Adriano Piattelli, Morena Petrini

**Affiliations:** 1 Department of Medical, Oral and Biotechnological Sciences, University of Chieti-Pescara, 66100 Chieti, Italy; simonetta.dercole@unich.it (S.D.); gio.iezzi@unich.it (G.I.); 2 Department of Dental Materials and Prosthodontics, School of Dentistry of Ribeirão Preto, University of São Paulo, São Paulo 05508-070, Brazil; tatianedotta@usp.br; 3 Toxicology and Diseases Group (TDG), Pharmaceutical Sciences Research Center (PSRC), The Institute of Pharmaceutical Sciences (TIPS), Tehran University of Medical Sciences, Tehran 1416634793, Iran; m.r.farani@ut.ac.ir; 4 Department of Materials Engineering-Tissue Engineering Najafabad Branch, Islamic Azad University, Isfhan 6134937333, Iran; niloofar.etemadi@yahoo.com; 5 Private Practice, San Vendemiano-Conegliano, 31020 Treviso, Italy; luca.comuzzi@gmail.com; 6 School of Dentistry, Saint Camillus International University for Health Sciences (Unicamillus), 00131 Rome, Italy; apiattelli51@gmail.com; 7 Fondazione Villa Serena per la Ricerca, 65013 Città Sant’Angelo, Italy; 8 Casa di Cura Villa Serena, 65013 Città Saint’Angelo, Italy

**Keywords:** implant-abutment connections, bacterial contamination, bacterial microleakage

## Abstract

The objective of this study is to evaluate, in vitro, the microleakage of bacteria of 3 different implant connections for a period of 14 days. 60 dental implants (AoN) (*n* = 20) were distinguished into three groups, accordingly to the type of connection: External Hexagon (EH), Internal Hexagon (IH), and Cone Morse (CM) connection. All implants were inserted and fixed on sterile special vinyl support. Ten fixtures for each group were inoculated in the internal platform with 1.0 μL of *Streptococcus oralis* (SO) and the other ten with the same amount of *Pseudomonas aeruginosa* (PA). The penetration of bacterial suspension into the surrounding solution was determined by the observation of the turbidity of the broth. Five implants for each sub-group were randomly observed at SEM, to verify the correct fitting of the abutments. Considering the total of the samples analyzed, CM showed significantly lower bacterial contamination, with respect to IH. In particular, bacterial contamination was found in 45%, 55%, and 20% of EH, IH, and CM, respectively. Analyzing results for the type of inoculated bacteria, *P. aeruginosa* showed a higher ability to contaminate all the connections, with respect to *S. oralis*.

## 1. Introduction

Failure in implant dentistry can be caused by several factors, such as surgical trauma, structural design, overload, peri-implantitis, periosteal reflection, the autoimmune response of the host, type of tightening (torque or pre-torque), presence of a microgap between implant and abutment and consequent bacterial microleakage [1,2,3].

Several studies have reported the penetration of bacteria into the cavities and gaps generated by the presence of microgap at the implant-abutment interface, causing a bacterial reservoir that can interfere with the health of the peri-implant tissue, causing inflammation and bone loss [3,4,5,6,7]. The size and shape of bacteria can be very variable: *Cocci* are spherical with a diameter of 1–3 µm; rods, comprehend very small bacteria like *Pelagibacter ubique* (diameter 0.2 µm; length 0.5 µm), *Escherichia coli* (diameter 1–2 µm; length 1–8 µm), and relatively big ones, like Epulopiscium fishelsoni (diameter 40–80 µm; length 250–600 µm) [8].

Some bacterial species are considered great colonizers of the implant surface and have been found in periimplantitis lesions. *Streptococcus oralis* (*S. oralis*) is an oral commensal organism, a member of the mitis group of viridans streptococci, a Gram-positive bacterium, and facultative aerobic. It is considered an opportunistic human pathogen, whose size ranges from 1 to 2 µm. *Pseudomonas aeruginosa* (*P. aeruginosa*) is a Gram-negative bacterium, aerobic/facultative anaerobe, a rod-shaped bacterium with unipolar motility. It is considered an opportunistic human pathogen, whose size ranges from 0.5 to 1 µm [9,10]. 

Under load conditions, the implant abutment junction can increase, and can lead to rotation and micro-movement of the abutment and lead to reduced screw preload, loosening, bending, and fracture [11,12,13].

In an attempt to limit the microgap and increase the stability of the abutment, a variety of implant-abutment connection designs have been developed, which are basically classified into internal and external connections [11]. The external connection usually has an external hexagon above the implant platform. And the internal connection can be further divided into passive joint or flat-to-flat systems (such as triangles, hexagons, and octagons) and conical interfaces or Cone Morse [14,15]. Combined implant-abutment connections are characterized by the combination of geometric features that provide antirotational and prosthetic positioning properties [15]. Even when the implant and the abutment are correctly connected, microleakage produced by a microcap can be generated, allowing the passage of acids, enzymes, bacteria, and/or their metabolic products [3,16,17].

The external connection is the one with the highest chance of bacterial microleakage, according to studies [18,19,20]. Others found no statistical differences in bacterial penetration into the implant/abutment complex between internal connections such as hexagons and Cone Morse [21,22,23]. On the other hand, other researchers have shown that bacterial species from human saliva penetrated internal hexagonal connection implants significantly more than the Cone Morse connection [20,24,25].

According to Baggi et al. [26], although the abutments were connected to the implants with the recommended torque, the geometry of some systems still permitted the passage of microorganisms. This is probably due to the different degrees of tolerance and different interface geometries that different implant systems and brands allow.

Indeed, the literature is still uncertain about such findings. Thus, the purpose of this in vitro study was to evaluate the microleakage of two bacterial species with different diameters at implant-abutment (I-A) interfaces of 3 different implant connections, for a period of 14 days. Applying a classical methodology in the measurement of bacterial infiltration, as well as comparing a bacterial species already studied, in vitro, in previous experiments, (*P. aeruginosa*), with one not yet analyzed (*S. oralis*).

## 2. Materials and Methods

A total of 60 dental implants AON (Grisignano di Zocco, Italy), 3.30 mm (diameter) × 11.5 mm (length) implants were used in this in vitro study:

- 20 with a screw-retained External Hexagon connection (EH)
- 20 with a screw-retained Internal Hexagon connection (IH)
- 20 with a Cone Morse taper internal connection with a screw-retained (CM).

All implants and prosthetic components were standard manufactured sterile samples. All other materials utilized in the experiment were sterilized inside surgical bags with the use of an autoclave. 

All procedures were performed under laminar flow in absolute sterility, by using components that were previously sterilized by the Manufacturer.

**Figure 1:**
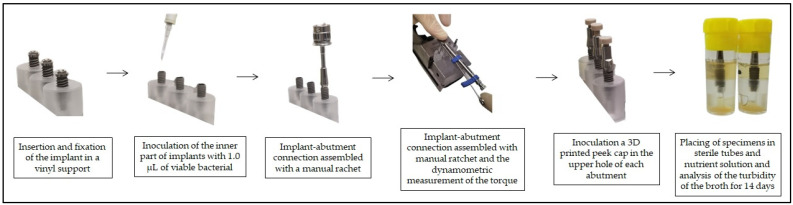
Study diagram.

**Figure 2:**
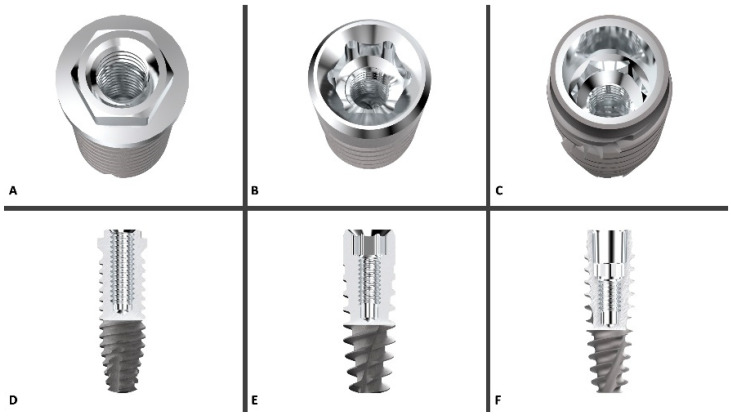
3D rendering of the three implant platforms compared in this study ((**A**) = External Hexagone, (**B**) = Internal Hexagone, (**C**) = Cone Morse), with the relative longitudinal section of the implant/abutment connection ((**D**) = EH, (**E**) = IH, (**F**) = CM). Courtesy of AON (Grisignano di Zocco, Italy).

To improve the explanation of the study and facilitate the reader’s understanding, below is a diagram with the stages of the experiment (Figure 1), and a schematic of the three different implant connections (Figure 2).

**Figure 3:**
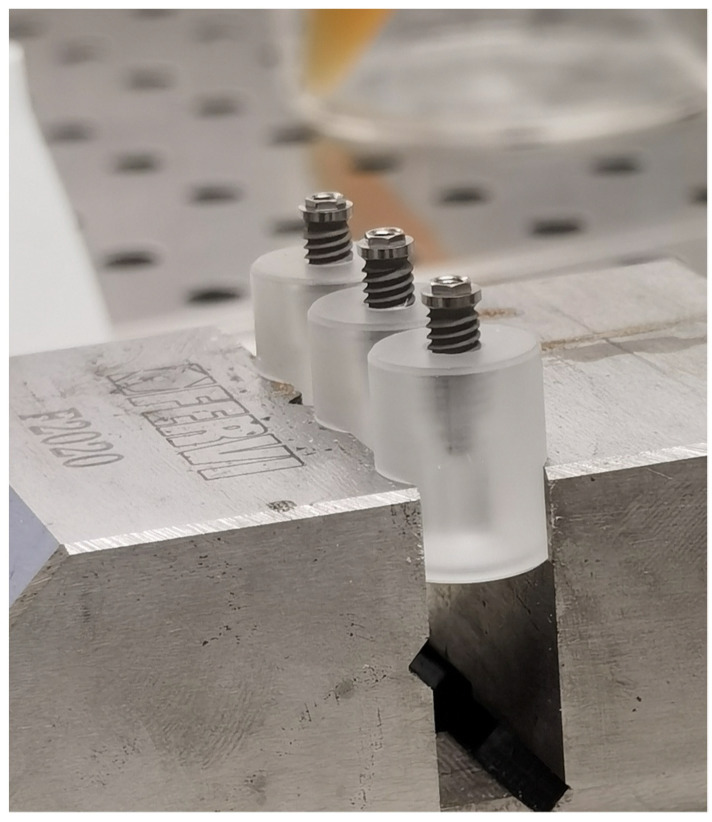
Insertion and fixation of the implant in a vinyl support.

In brief, each implant was inserted and fixed on a sterile special vinyl support apposite produced for this in vitro study. The body of the fixtures was submersed in the base by living the more occlusal threads free. In order, to avoid any movement of the base during the screwing of the abutment, each base was fixed in a morse, as shown in Figure 3.

### 2.1. Bacterial Inoculation

Pure cultures of *Streptococcus oralis* CH 05 and *Pseudomonas aeruginosa* ATCC 15,442 were used for implant inoculation. For the preparation of the bacterial suspension, the tested microorganisms *S. oralis* and *P. aeruginosa* were first plated onto fresh trypticase soya and cetrimide agar, respectively, incubated for 24 h at 37 °C and standardized at optical density OD_600_ 0.125 [7,27,28,29]. 20 specimens of each group were tested in the microbiological experiment.

**Figure 4:**
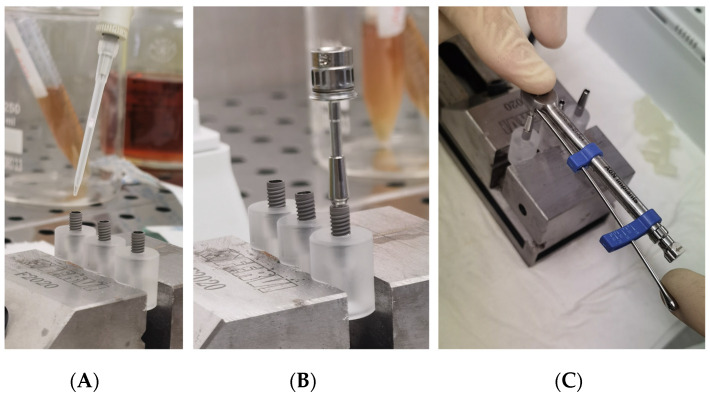
(**A**) Inoculation of the inner part of implants with 1.0 µL of viable bacterial; (**B**) An implant-abutment connection assembled with a manual rachet; (**C**) An implant-abutment connection assembled with a manual ratchet and the dynamometric measurement of the torque.

Each group was divided into two sub-group, and each fixture was inoculated with 1.0 μL of different standardized broth cultures (Figure 4A):

- Subgroup SO (10 fixtures for each group): inoculated with *S. oralis* CH 05
- Subgroup PA (10 fixtures for each group): inoculated with *P. aeruginosa* ATCC 15442

### 2.2. Abutment Connection

In all cases, after the implant inoculation, the abutment was carefully connected to the implant, according to the manufacturer’s protocol, without touching the outer surface of the implant and while using sterile gloves.

**Figure 5:**
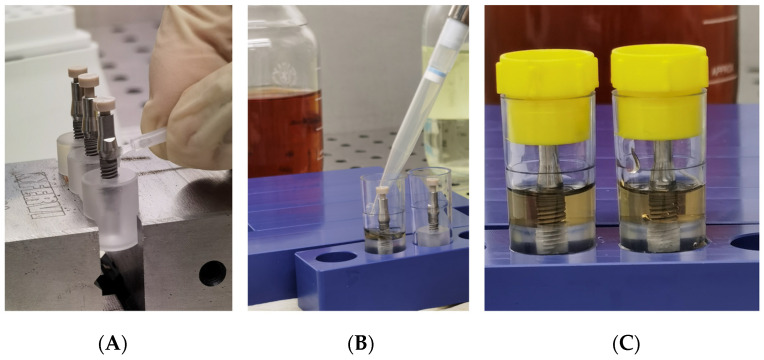
(**A**) Inoculation of a 3D printed peek cap in the upper hole of each abutment; (**B**) Placing of specimens in sterile tubes and nutrient solution; (**C**) Closure of tubes with cap.

A specific dynamometric manual ratchet was used to screw the abutments with the optical torque, as suggested by the Manufacture (Figure 5B,C).

In particular, the following torques were used:

- External Hexagon: insert the passing screw, and tighten it to 30 Ncm
- Internal Hexagon: insert the passing screw, and tighten it to 30 Ncm
- Cone Morse: Insert the conometric activation key (AoN), tighten up to 35 Ncm, remove the key with a reverse torque, insert the passing screw, and tighten it to 25 Ncm.

During the screwing, the abutments were managed only by touching with sterilized pliers.

After the abutment connection, a 3D-printed peek cap was inserted in the upper hole of each abutment, to prevent the passage of bacteria, from this upper interface (Figure 5A).

As a positive control, 2 identified test tubes were used with only nutrient solution and inoculated with 1.0 μL of *S. oralis* and *P. aeruginosa*, respectively. They showed bacterial growth with solution cloudiness, and this confirmed the viability of the microorganisms throughout the experiment. As a negative control, 2 identified test tubes were used with only sterile nutrient solution. This was confirmed by the transparency of the solution and conventional microbial culturing techniques.

Subsequent to inoculation and abutment connection, the assembled components were totally immersed for 1 min inside the nutrient solution in a rolling motion for evaluation of inadvertent contamination of the external surface. Tubes with a cloudy broth (indicative of colonization/contamination of the outer parts of the implant) were excluded from further observation after evaluation of bacterial growth in plates. Then, the specimens were placed into sterile tubes and the volume of nutrient solution required in the test vials was determined exactly for each implant system, so that the fluid level remained just above the I-A interfaces (Figure 5B). Then all tubes were closed with a cap and then left for observation (Figure 5C).

**Figure 6:**
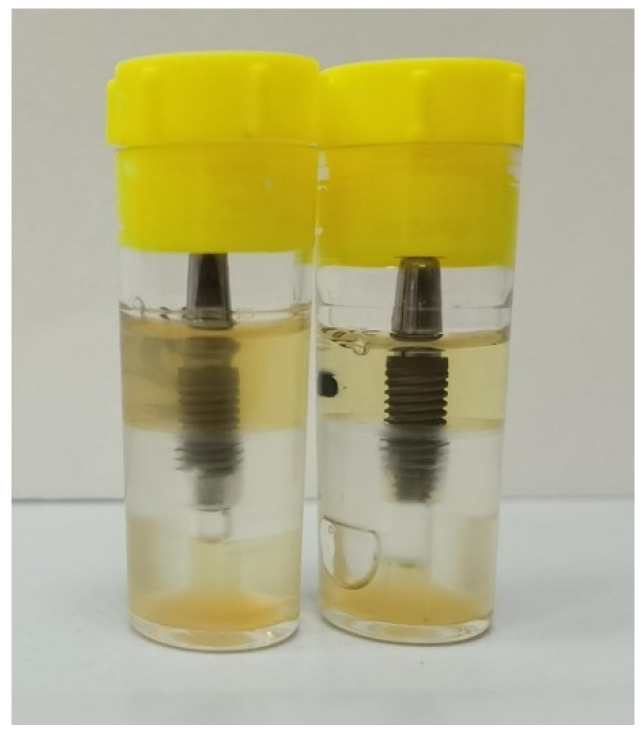
Samples placed into the nutrient solution during the follow-up. **Left** = turbidity of the broth as a sign of bacterial penetration; **Right** = no contamination.

All the vials containing the assemblies, the test tubes used as external contamination control, the test tubes used as a positive control, and the test tubes used as negative control were incubated at 37 °C under aerobic conditions. They were maintained for 14 days, and the culture broth in the vials containing the assemblies was replaced every 4 days. The possible penetration of bacterial suspension into the surrounding solution was determined by the visual observation of the turbidity of the broth (Figure 6). The samples were checked daily, and any presence or absence of turbidity was recorded. Such leakage caused bacterial colonization and resulted in a cloudy solution, 1 μL of the solution was analyzed with a gram stain and by colony morphology in trypticase agar plates (for *S. oralis*) and cetrimide agar (for *P. aeruginosa*), incubated at 37 °C for 24 h to confirm the purity of the microorganism which had been inoculated in the inner part of the implant and determining the presence of *S. oralis* and *P. aeruginosa*, respectively.

### 2.3. SEM Analysis

A Phenom ProX scanning electron microscope (Phenom-World BV, Eindhoven, The Netherlands) was utilized with the Element Identification (EID) package (Phenom ProSuite Software, Phenom-World B.V., Eindhoven, The Netherlands). 5 implants for each sub-group were observed at SEM at 240× of magnification at 15 Kv, to verify the correct fitting of the abutments [30]. The purpose of these images was to verify qualitatively the complete fitting between the prefabricated components.

So, before SEM observation, the samples were decontaminated, disinfected, and sterilized, to avoid the risk of microbial contamination during the SEM observation.

### 2.4. Statistical Analysis

The total number of implants per group exhibiting bacterial colonization of the microgap was reported.

The evaluation of homogeneity of the groups was analyzed using the Levene test. The differences between the groups were statistically analyzed using the analysis of variance (ANOVA) and the Fisher’s Least Significant Difference (LSD). Statistically significant differences were considered to be a *p*-value < 0.05. The statistical software used to run these tests was SPSS Statistics for Windows, version 21 (IBM SPSS Inc., Chicado, IL, USA).

## 3. Results

**Figure 7:**
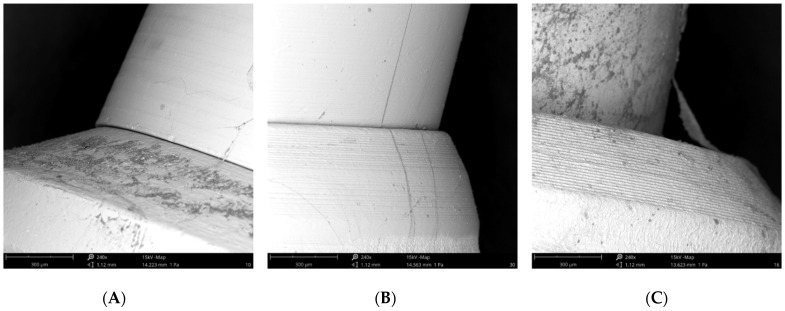
(**A**) SEM image of the External Hexagon connection; (**B**) SEM image of the Internal Hexagon connection; (**C**) SEM image of the Cone Morse connection.

All SEM observations confirmed the correct fitting of the abutments in the implant connections (Figure 7).

Table 1 shows the percentage of connections showing bacterial contamination in the nutrient solution over the 14-day observation period. At the beginning of the study, both groups of implants and abutments analyzed were equally sterile, and therefore comparable from a statistical point of view. In total, CM showed a significant lower contamination, respect IH (*p* = 0.025). 

*Pseudomonas aeruginosa* showed a higher ability to contaminate all implant connections, respect *Streptococcus oralis*.

In particular, in the EH, 30% bacterial contamination was found in I-A assemblies seeded with *S. oralis* and 60% seeded with *P. aeruginosa*.

And in the Internal Hexagon implants (IH), 40% bacterial contamination was found in I-A assemblies seeded with *S. oralis* and 70% seeded with *P. aeruginosa*. And in the Cone Morse implants (CM), 40% bacterial contamination was found in I-A assemblies seeded with *P. aeruginosa* while none for *S. oralis.* Although a lower contamination percentage was found on CM, with respect to other groups, no statistically significant differences were recorded for *P. aeruginosa* and *S. oralis* contamination, in the sub-group analysis.

## 4. Discussion

The use of implants in oral rehabilitation has become a great option for the treatment of partially or totally edentulous patients. However, microgap formation at implant-abutment interfaces can act as a bacterial reservoir, and tissue inflammation and bone loss may occur, leading to injury or failure of the implants, and impacting the biological success of implant treatment [2,4,31].

The potential colonization of the microgap is probably related to multifactorial conditions, i.e., the imprecise fit between the implant components and improper male-female adaptation, imprecise machining of implant parts, the torque forces used to connect the components leading to part distortion, and the loading forces when the implants are in function [1,28]. Analyzing qualitative SEM images at 240x magnification, we can verify the correct fitting of the abutments in the implant connections.

Several studies defend the possibility of a lower bacterial infiltration in Morse Cone connections compared to external and internal hexagon connections. Verdugo et al. [18], determined that Morse taper connection implants showed lower levels of microleakage than external connection implants. In the narrative review of Lauritano et al. [19], after evaluating 55 articles, they found that conical connections were better in relation to bacterial sealing. D’Ercole et al. [28], reported high permeability to bacterial leakage of screw-retained abutment connections, and the lower infiltration rates, although not significantly, of Cone Morse taper internal connections.

This, in fact, is also shown in our study, since Morse Cone connections proved to be more effective in bacterial sealing, presenting only a 20% of contamination, compared to the EH 45%, and IH 55%.

Due to its self-locking characteristics and high stability with the absence of micromovements between the parts during function, the Morse Cone taper internal connection seems to be able to resist the penetration of bacteria more [32]. According to Teixeira et al. [23], the lateral loads are resisted mainly by the tapered interface, which prevents the abutment from tilting off. This mechanism, referred to as positive or geometric locking, is responsible for protecting the abutment threads from excessive functional loading. There is no possibility of tilting about a single point or small area. Despite these findings, studies like that of Teixeira et al. [23], observed the bacterial leakage through the implant/abutment interface in Morse taper and internal-hexagon implants.

In the present study, although the implant/abutment assemblies were assembled according to the manufacturer’s recommendations, microleakage of the selected microorganisms (*S. oralis* and *P. aeruginosa*) nevertheless occurred. *Streptococcus oralis* is highly abundant at implant sites and human gingival fibroblasts and human gingival epithelial cells, are the main cell types in peri-implant tissue. *Pseudomonas aeruginosa* has the ability to form biofilms and can be present in the bloodstream and periodontal infections. The use of those bacteria seems relevant for in vitro studies because these microorganisms have been found in periimplantitis lesions [17,27,28,32,33]. These bacteria were chosen over other known periodontal pathogens because they are easy to culture and because of their reduced size, their permeability through the microgap of the I-A interface, and their common residence in the peri-implant area [34].

Since the size of the mean microgap from the abutment to the implant junction has been reported to be 1 to 49 μm [8], in fact, bacterial infiltration could occur, mainly in groups conditioned to *P. aeruginosa*, due to their smaller size.

Despite the type of connection and even when the implant and the abutment are correctly connected, microleakage produced by a microcap can be generated, allowing the passage of bacteria and metabolic products, and may result in soft tissue inflammation, constituting a risk to the stability and clinical success of the implants. Thus, the improvement of implant dentistry materials is expected, so that the annihilation of the microbial passage in implant-abutment connections occurs. And still, new studies should be developed to better study bacterial infiltration with such bacteria, as well as performing new microbiological analyzes and measuring the size of the microgap.

## 5. Conclusions

Within the limits of this study, the following conclusions were drawn:

1. The in vitro leakage of *Pseudomonas aeruginosa* through the abutment/implant interface occurred at both types of interface connections tested-Cone Morse, external and internal hexagon- and with more intensity than *Streptococcus oralis.*
2. Less bacterial leakage and a lower rate of infiltration in Cone Morse connections when compared to Internal Hexagon connections.

## Figures and Tables

**Table 1:** Bacterial leakage in implants with different implant-abutment connections inoculated with *Streptococcus oralis* and *Pseudomonas aeruginosa* over a 14-day observation period.

Implants	Bacterial Species	Contamination with Different Species %	Total Contaminations%
EH	*S. oralis* (SO)	30%	45%
	*P. aeruginosa* (PA)	60%	
IH	*S. oralis* (SO)	40%	55%
	*P. aeruginosa* (PA)	70%	
CM	*S. oralis* (SO)	---	20%
	*P. aeruginosa* (PA)	40%	

## Data Availability

All datasets generated for this study are included in the article.
